# Mutual-information based optimal experimental design for hyperpolarized $^{13}$C-pyruvate MRI

**DOI:** 10.1038/s41598-023-44958-y

**Published:** 2023-10-23

**Authors:** Prashant K. Jha, Christopher Walker, Drew Mitchell, J. Tinsley Oden, Dawid Schellingerhout, James A. Bankson, David T. Fuentes

**Affiliations:** 1 Oden Institute for Computational Engineering and Sciences, The University of Texas at Austin, Austin, TX 78712 USA; 2 Department of Imaging Physics, MD Anderson Cancer Center, Houston, TX 77320 USA

**Keywords:** Functional magnetic resonance imaging, Computational science, Computational models, Image processing

## Abstract

A key parameter of interest recovered from hyperpolarized (HP) MRI measurements is the apparent pyruvate-to-lactate exchange rate, $k_{{\textrm PL}}$, for measuring tumor metabolism. This manuscript presents an information-theory-based optimal experimental design approach that minimizes the uncertainty in the rate parameter, $k_{{\textrm PL}}$, recovered from HP-MRI measurements. Mutual information is employed to measure the information content of the HP measurements with respect to the first-order exchange kinetics of the pyruvate conversion to lactate. Flip angles of the pulse sequence acquisition are optimized with respect to the mutual information. A time-varying flip angle scheme leads to a higher parameter optimization that can further improve the quantitative value of mutual information over a constant flip angle scheme. However, the constant flip angle scheme, 35 and 28 degrees for pyruvate and lactate measurements, leads to an accuracy and precision comparable to the variable flip angle schemes obtained from our method. Combining the comparable performance and practical implementation, optimized pyruvate and lactate flip angles of 35 and 28 degrees, respectively, are recommended.

## Introduction

The potential of hyperpolarized (HP) $^{13}$C-Pyruvate magnetic resonance imaging (MRI) to characterize cancer biology, predict progression, and monitor early responses to treatment is well known (e.g.,^1–9^). Ongoing studies in prostate, brain, breast, liver, cervical, and ovarian cancer^1, 3, 4^ have shown that HP $^{13}$C-Pyruvate MRI is safe and effective. One of the central aspects of HP-MRI that make it appealing is the elevated chemical conversion of pyruvate to lactate, even in the presence of oxygen, via lactate dehydrogenase (LDH), also referred to as the Warburg effect^10, 11^. The higher production of lactate has been shown to correlate with disease presence, the aggressiveness of the disease (e.g., cancer and inflation), and response to therapy. The rate of pyruvate-to-lactate exchange ($k_{{\textrm PL}}$) is a crucial parameter of interest in locating aggressive disease and assessing the biological state of the tissue. HP-MRI presents a unique opportunity to observe tumor metabolism *in vivo*^2, 3, 5, 6^ and use this information to make inferences about tumor aggressiveness and response to therapy. However, a recent white paper^3^ highlights the need for further development of spatial, temporal, and spectral encoding strategies that minimize uncertainty while maximizing the efficiency of HP-MRI. An example of uncertainty is the variability of the reported HP measurements in the literature^12–14^. In the present work, we develop an information-theory-based approach to determine the optimal MRI design parameters, such as flip angles, with a goal of reducing the uncertainty in the recovered rate parameter, $k_{{\textrm PL}}$.

The physics of the dynamic nuclear polarization (DNP) method that enables MR imaging of HP $^{13}$C-pyruvate is described in^3, 5^. The time history of pyruvate and lactate magnetization within the imaging voxels constitutes the data of interest. Together with a pharmacokinetic HP-MRI model, pyruvate and lactate magnetization data are employed to recover the pyruvate-to-lactate apparent exchange rate, $k_{{\textrm PL}}$; e.g.,^2, 15, 16^. Accuracy and uncertainty in the recovered rate parameter depend on the data’s information content and the pharmacokinetic model’s fidelity. This work aims to determine the MRI design parameters that increase the information content in the data and reduce the uncertainty in the rate parameter. The mutual information (MI) between the data and critical model parameters is utilized toward this goal. Information theory and, specifically, mutual information provides a rigorous mathematical framework for optimizing acquisition protocols to improve reproducibility.

In this work, data to verify the reduction in uncertainty of recovered rate parameter $k_{{\textrm PL}}$ when using optimal design parameters is generated synthetically from the pharmacokinetic model. Different signal-to-noise ratios are considered in generated noisy data to analyze the uncertainty in the recovered $k_{{\textrm PL}}$ for various signal-to-noise ratios. The optimal design parameters is shown to reduce uncertainty in the pyruvate-to-lactate apparent exchange rate, $k_{{\textrm PL}}$. The codes and relevant files to reproduce the results will be publicly available in the following GitHub repository: https://github.com/prashjha/HyperpolarizedMRI.

### Related works

Semi-quantitative metrics such as the ratio of the integrals of the lactate-to-pyruvate signals (area under the curve, AUC) are often preferred due to their simplicity. However, the lactate-to-pyruvate ratio is biased by HP pyruvate in blood vessels that are inaccessible to enzymes that mediate conversion from HP pyruvate to lactate. For example, if the vascular blood volume decreases by 10 percent in a region of a tumor, the lactate-to-pyruvate ratio could increase even if the true metabolic state of cells does not change. The lactate-to-pyruvate ratio is also affected by the excitation scheme. Small flip angles consume less pyruvate magnetization and permit the signal pool to remain longer for the conversion to lactate. The potential for variability is realized in the literature. In applications to brain cancer (glioma) Grist^12^ reports a lactate to pyruvate ratio of .25 ± .08 and .22 ± .06 in white matter and gray matter, respectively. Lactate-to-pyruvate ratios greater than 1.0 are reported in^13^ in gray matter. Lactate-to-pyruvate ratios in white matter of 0.43 ± 0.14 were reported in^14^.

Various strategies have been proposed to optimize the HP acquisition in terms of the variability and accuracy of the pyruvate to lactate conversion rate measurements, $k_{{\textrm PL}}$. Walker et al.^15^ utilized a two-species kinetic model and compared variable excitation angle acquisitions and conventional constant excitation angle acquisitions. The variable excitation scheme was obtained from a recursive relation that depletes the signal at the final acquisition^17^. Optimization strategies included maximization of the lactate signal or maintaining a constant signal. Either constant excitation angle or variable excitation angles that attempt to maximize total signal of the acquisition, as opposed to maintaining a constant signal level, were seen to produce the best recovery in terms of accuracy and repeatability. Larson et al.^18^ developed a novel kinetic modeling approach that did not require a manual input forcing function for the governing two-species kinetic model equations. The ‘inputless’ method required no assumptions regarding the input function and was compared to a tradition kinetic model that manually identified the known input signal. Assuming time-varying excitation angles, the ‘inputless’ method was seen to provide no loss in accuracy and precision over the classical method. Maidens et al.^19^ develop a Fisher information approach for determining the optimal time varying pulse sequence and demonstrate a decrease in $k_{{\textrm PL}}$ uncertainty when compared with strategies that maximize the signal SNR (Signal-to-noise ratio). In^20^, the OED formulation for magnetic resonance fingerprinting based on the Cramér-Rao bound is presented. Cramér-Rao bound was used for optimal estimation of parameters in Bloch equation in^21^.

The information theoretic approach developed in this work is an extension of optimal experimental design approaches that use the Fisher information matrix and the Cramér-Rao bound as a lower bound on the variance of unbiased estimators^16, 19–28^. Indeed, the de Bruijn identity^29^ provides a direct connection between derivatives of our entropy calculations and the Fisher information matrix. However, optimizing the Fisher information matrix requires estimates of the unknown tissue parameters, such as ${\textrm T}_1$ relaxation losses and pyruvate-to-lactate conversion rate (parameter to be recovered from the data), to calculate the Fisher information. The Fisher information must be iteratively re-optimized as more accurate estimates of the tissue properties are obtained. In contrast, the present approach provides a mathematical framework to directly include the tissue parameter uncertainty in the mutual information and considers a range of tissue parameters (determined by the input probability distributions) to calculate and optimize the mutual information.

## Methods

### Hyperpolarized (HP) MRI model

Consider a tissue domain within which different constituents evolve depending on the local environment which includes, for example, extravascular (interstitial) and vascular. In this work, two spatial compartments, namely, extravascular and vascular, each containing HP pyruvate and lactate and complement of these two constituents, are considered. The model employed is based on^2, 15^ and accounts for ${\textrm T}_1$ relaxation loss, signal loss due to excitation at each scan, and pyruvate-to-lactate and lactate-to-pyruvate exchanges. We expect a dynamic, spatially, and spectrally localized data from the HP MR data acquisition such that the spatially invariant model may represent the data collection on a voxel-by-voxel basis.

Consider a tissue domain $\Omega _{{\textrm cell}}$ of volume $|\Omega _{{\textrm cell}}|$ in units of cm$^3$. Assuming $\Omega _{{\textrm cell}}$ is small enough that the spatial variation of hyperpolarized agents in $\Omega$ can be ignored, we let $\bar{{\textrm c}}_{{\textrm P}}(t)$ and $\bar{{\textrm c}}_{{\textrm L}}(t)$ denote the relative density of HP pyruvate and lactate, respectively, at time *t* (in units of s). Here, the relative density of species ${\textrm A}\in \{{\textrm P}, {\textrm L}\}$ is defined as the ratio of the density of species $\rho _{{\textrm A}}$ and the total density $\rho$, i.e., $\bar{{\textrm c}}_{{\textrm A}} = \rho _{{\textrm A}} / \rho$. Then the total mass of HP pyruvate and lactate are $\rho |\Omega _{{\textrm cell}}| \bar{{\textrm c}}_{{\textrm P}}(t), \rho |\Omega _{{\textrm cell}}| \bar{{\textrm c}}_{{\textrm L}}(t)$, respectively, $\rho$ being the mass density (g/cm$^3$) of the continuum mixture. Discrete times at ${\textrm N}$ scans are denoted by $t_k$, $1\le k \le {\textrm N}$; $\theta ^k_{{\textrm P}}$ and $\theta ^k_{{\textrm L}}$ are flip angles in ${k}^{\text {th}}$ scan whereas ${\textrm TR}_k = t_k - t_{k-1}$, $k > 1$, are repetition times and ${\textrm TR}_1 = 0$. With $\bar{\varvec{{\textrm c}}}= (\bar{{\textrm c}}_{{\textrm P}}, \bar{{\textrm c}}_{{\textrm L}})^T$, the HP pyruvate and lactate available for measurement at the ${(k+1)}^{\text {th}}$ scan, $k \ge 1$, are given by^15^

1 $$\begin{aligned} \bar{\varvec{{\textrm c}}}(t_{k+1}) = \exp \left[ {\textrm TR}_{k+1} \varvec{{\textrm A}} \right] \varvec{{\textrm C}}^k \bar{\varvec{{\textrm c}}}(t_k) + \frac{ k_{{\textrm ve}}}{\nu _{\textrm e}} \int _{t_k}^{t_{k+1}} \exp \left[ (t_{k+1} - \tau ) \varvec{{\textrm A}} \right] \varvec{{\textrm VIF}}(\tau ) d\tau , \end{aligned}$$

where the matrix $\varvec{{\textrm A}}$ accounts for $T_1$ relaxation losses and pyruvate-lactate exchanges

2 $$\begin{aligned} \varvec{{\textrm A}} = \begin{bmatrix} -\frac{1}{{\textrm T}_{1,{\textrm P}}} - k_{{\textrm PL}}-\frac{ k_{{\textrm ve}}}{\nu _{\textrm e}} &{} k_{{\textrm LP}}\\ k_{{\textrm PL}}&{} -\frac{1}{{\textrm T}_{1,{\textrm L}}} - k_{{\textrm LP}} \end{bmatrix} . \end{aligned}$$

Here ${\textrm T}_{1,{\textrm P}}, {\textrm T}_{1,{\textrm L}}$ denote the ${\textrm T}_1$ relaxation times (s), $k_{{\textrm PL}}, k_{{\textrm LP}}$ pyruvate-to-lactate and lactate-to-pyruvate exchange rates (s$^{-1}$), $k_{{\textrm ve}}$ vascular-tissue exchange rate (s$^{-1}$), and $\nu _{\textrm e}$ the extravascular volume fraction. In (1), $\varvec{{\textrm C}}^k$ denotes the matrix that takes into account the loss of signal due to excitation at ${k}^{\text {th}}$ scan:

3 $$\begin{aligned} \varvec{{\textrm C}}^k = \begin{bmatrix} \cos (\theta _{{\textrm P}}^k) &{} 0 \\ 0 &{} \cos (\theta _{{\textrm L}}^k) \end{bmatrix} . \end{aligned}$$

Lastly, $\varvec{{\textrm VIF}}= \varvec{{\textrm VIF}}(t)$ is the vascular input function (dimensionless). Empirically determined blood flow through nonbranching vessels have been shown to correspond to a gamma variate input function^30^. Thus, $\varvec{{\textrm VIF}}$ is taken to be

4 $$\begin{aligned} \varvec{{\textrm VIF}}(t) = \begin{bmatrix} \sigma _{{\textrm P}} \gamma (t - \bar{t}_0, \alpha _{{\textrm P}}, \beta _{{\textrm P}}) \\ 0 \end{bmatrix}, \end{aligned}$$

where $\sigma _{{\textrm P}}, \alpha _{{\textrm P}}, \beta _{{\textrm P}}$ are constants, and $\gamma$ denotes a gamma probability density function given by

5 $$\begin{aligned} \gamma (t, a, b) = \frac{1}{b^a \Gamma (a)} t^{a-1} \exp \left[ -\frac{t}{b}\right] . \end{aligned}$$

The constant $\bar{t}_0$ is associated with bolus arrival time and is treated as one of the uncertain model parameters. Constants $\sigma _{{\textrm P}}$ and $\alpha _{{\textrm P}}$ are dimensionless while $\beta _{{\textrm P}}$ is in units of seconds.

In (1), the parameters can be gathered in two different classes: 1) model parameters that depend on the tissue domain and specific voxel and includes  $\mathcal {P} = ({\textrm T}_{1, {\textrm P}}, {\textrm T}_{1, {\textrm L}}, k_{{\textrm PL}}, k_{{\textrm LP}}, k_{{\textrm ve}}, \nu _{\textrm e}, \bar{t}_0)$, and 2) HP-MRI design parameters such as repetition times and flip angles $\mathcal {K}= (\{{\textrm TR}_i\}_{i=2}^{\textrm N}, \{\theta _{{\textrm P}}^i\}_{i=1}^{\textrm N}, \{\theta _{{\textrm L}}^i\}_{i=1}^{\textrm N})$. For simplicity, the parameters ${\textrm T}_{1, {\textrm P}}, {\textrm T}_{1, {\textrm L}}, \nu _{\textrm e}, k_{{\textrm LP}}$ are assumed to be known and fixed so that $\mathcal {P}= (k_{{\textrm PL}}, k_{{\textrm ve}}, \bar{t}_0)$. The default values of model and design parameters are provided in Tables 1and 2, respectively.

#### The total signal

The magnetization intensity of constituent ${\textrm A}$, ${\textrm A}\in \{{\textrm P}, {\textrm L}\}$, at ${k}^{\text {th}}$ scan is assumed to be proportional to the mass of the constituent in $\Omega _{{\textrm cell}}$, i.e., there is a constant ${\textrm C}$ such that ${\textrm C}\rho \bar{{\textrm c}}_{{\textrm P}}(t_k) |\Omega _{{\textrm cell}}|$ and ${\textrm C}\rho \bar{{\textrm c}}_{{\textrm L}}(t_k) |\Omega _{{\textrm cell}}|$ are the total magnetization of pyruvate and lactate, respectively. Without loss of generality, ${\textrm C}$ is such that ${\textrm C}\rho |\Omega _{{\textrm cell}}| = 1$. In this case, $\bar{{\textrm c}}_{{\textrm P}}(t_k), \bar{{\textrm c}}_{{\textrm L}}(t_k)$ are the total magnetizations, $\sin (\theta _{{\textrm P}}^k) \bar{{\textrm c}}_{{\textrm P}}(t_k)$ and $\sin (\theta _{{\textrm L}}^k) \bar{{\textrm c}}_{{\textrm L}}(t_k)$ are the measured signal, and $\cos (\theta _{{\textrm P}}^k) \bar{{\textrm c}}_{{\textrm P}}(t_k)$ and $\cos (\theta _{{\textrm L}}^k) \bar{{\textrm c}}_{{\textrm L}}(t_k)$ are the signals remaining for the next measurements, respectively.

The signals measured at the ${k}^{\text {th}}$ scan are, see^15^,

6 $$\begin{aligned} \varvec{{\textrm s}}^k = \begin{bmatrix} \sin (\theta _{{\textrm P}}^k) &{} 0 \\ 0 &{} \sin (\theta _{{\textrm L}}^k) \end{bmatrix} \left( \nu _{\textrm e}\bar{\varvec{{\textrm c}}}(t_{k}) + (1-\nu _{\textrm e}) \varvec{{\textrm VIF}}(t_{k}) \right) = \begin{bmatrix} \sin (\theta _{{\textrm P}}^k)\left( \nu _{\textrm e}\bar{{\textrm c}}_{{\textrm P}}(t_k) + (1-\nu _{\textrm e}) \varvec{{\textrm VIF}}_1(t_k) \right) \\ \sin (\theta _{{\textrm L}}^k)\left( \nu _{\textrm e}\bar{{\textrm c}}_{{\textrm L}}(t_k) + (1-\nu _{\textrm e}) \varvec{{\textrm VIF}}_2(t_k) \right) \end{bmatrix} , \end{aligned}$$

i.e., one only measures the $\sin (\theta _{{\textrm P}}^k)$ and $\sin (\theta _{{\textrm L}}^k)$ fractions of the magnetization leaving the $\cos (\theta _{{\textrm P}}^k)$ and $\cos (\theta _{{\textrm L}}^k)$ fractions for the next measurement. The total signal is the sum of the individual signals at different scans and is given by

7 $$\begin{aligned} \mathcal {G}= \mathcal {G}(\mathcal {K}, \mathcal {P}) = \sum _{k=1}^{\textrm N}\left( {\textrm s}_{{\textrm P}}^k + {\textrm s}_{{\textrm L}}^k \right) , \end{aligned}$$

where the dependence of $\mathcal {G}$ on the design parameters $\mathcal {K}$ and model parameters $\mathcal {P}$ is clear from (1) and (6). Note that the total signal depends on the entire history of the magnetization time evolution. The magnetization at the current acquisition depends on the HP signal available from the previous acquisition and the total signal is the sum over each time point. In general, the total signal is also proportional to the magnetization weighted by B1 sensitivity maps of the receive coils used for the acquisitions. Here, we are considering the HP data fits on a per pixel basis and assume that B1 variations are small across a given pixel.

**Table 1:** Default model parameters, $\mathcal {P}$, and remaining fixed model parameters used in initialization, optimization, and verification steps.

Parameter	Default value	Description
${\textrm T}_{1, {\textrm P}}, {\textrm T}_{1, {\textrm L}}$	30 s, 25 s	Relaxation times^31^
$k_{{\textrm PL}}$	0.15 s$^{-1}$	Pyruvate-to-lactate apparent exchange rate
$k_{{\textrm LP}}$	0 s$^{-1}$	Lactate-to-pyruvate apparent exchange rate
$\bar{t}_0$	4 s	Bolus arrival time for HP pyruvate
$\sigma _{\textrm P}, \alpha _{\textrm P}, \beta _{\textrm P}$	100, 2.5, 4.5 s	Parameters in the gamma function (4)
$k_{{\textrm ve}}$	0.05 s$^{-1}$	Vascular-extravascular exchange rate
$\nu _{\textrm e}$	0.95	Extravascular volume fraction

**Table 2:** Default design parameters, $\mathcal {K}$, used in initialization, optimization, and verification steps.

Parameter	Default value	Range	Description
${\textrm N}$	30	–	Number of HP-MRI scans
${\textrm TR}_k$	3 s	[1.5, 4.5]	Repetition times (for $k> 1$)
$\theta _{{\textrm P}}^k$	20 degrees	[0, 35]	Flip angles for HP pyruvate (for all *k*)
$\theta _{{\textrm L}}^k$	30 degrees	[0, 35]	Flip angles for HP lactate (for all *k*)

### Synthetic data

To verify the uncertainty reduction in $k_{{\textrm PL}}$ using the optimal design parameters, HP-MRI experiment is synthetically simulated using the model in (1) with the optimal design parameters and the control design parameters (default values listed in Table 2). Suppose $\mathcal {K}_{{\textrm S}}$ is the design parameter associated with some scenario ${\textrm S}$; ${\textrm S}= {\textrm default}$ correspond to the default design parameters, ${\textrm S}= {\textrm OED}_{{\textrm SNR}}$ correspond to the optimized design parameters for the specific ${\textrm SNR}$ value.

Using the pharmacokinetic model (1), “ground truth” (signals at ${\textrm N}$ scans) is generated using $\mathcal {K}_{{\textrm S}}$ design parameters and default model parameters listed in Table 1. The data (simulation results) is denoted by ${\textrm Y}_{{\textrm S}}$ and takes the form:

$$\begin{aligned} {\textrm Y}_{{\textrm S}} = \begin{bmatrix} {\textrm s}^1_{\textrm P}&{} {\textrm s}^1_{\textrm L}\\ {\textrm s}^2_{\textrm P}&{} {\textrm s}^2_{\textrm L}\\ \cdot &{} \cdot \\ {\textrm s}^{\textrm N}_{\textrm P}&{} {\textrm s}^{\textrm N}_{\textrm L} \end{bmatrix}, \end{aligned}$$

where, ${\textrm s}^i_{\textrm P}, {\textrm s}^i_{\textrm L}$ denote the pyruvate and lactate signals at ${i}^{\text {th}}$ scan, see (6). A total of 25 samples of noisy data for different values of SNR are considered. Noisy samples are computed as follows:

8 $$\begin{aligned} {\textrm Y}_{{\textrm noisy}, {\textrm S}, {\textrm SNR}_{{\textrm data}}} = {\textrm Y}_{{\textrm S}} + \sigma _{{\textrm s}}({\textrm SNR}_{{\textrm data}}) \begin{bmatrix} a_1 &{} b_1 \\ a_2 &{} b_2 \\ \cdot &{} \cdot \\ a_{\textrm N}&{} b_{\textrm N} \end{bmatrix} , \end{aligned}$$

where $\sigma _{\textrm s}({\textrm SNR}_{{\textrm data}})$, given in (21), is the amount of noise in measured signals that depends on the assumed SNR, ${\textrm SNR}_{{\textrm data}}$, and $a_j, b_j\sim \mathcal {N}(0,1)$ for each $j=1,2,..,{\textrm N}$. Since SNR in the data, ${\textrm SNR}_{{\textrm data}}$, is expected to vary with pixelwise location, a range of ${\textrm SNR}_{{\textrm data}}$ for different optimal design parameters is considered to comprehensively evaluate the accuracy and precision of the $k_{{\textrm PL}}$ parameter recovery. The noise values correspond to the previous SNR range considered: $\sigma _{\textrm s}({\textrm SNR}_{{\textrm data}})$ for ${\textrm SNR}_{{\textrm data}} \in \{2, 5, 10, 15, 20\}$.

### Mutual information based optimization of MR scan parameters

A major goal of this study is to formulate an optimization problem to determine the design parameters $\mathcal {K}= ({\{{\textrm TR}_i\}_{i=2}^{\textrm N}}, \{\theta _{{\textrm P}}^i\}_{i=1}^{\textrm N}, \{\theta _{{\textrm L}}^i\}_{i=1}^{\textrm N})$ such that the MRI measurements reduce uncertainty in the rate parameter, $k_{{\textrm PL}}$. Treating total signal, $\mathcal {G}$ defined in (7), as the data, and model parameters, $\mathcal {P}= (k_{{\textrm PL}}, k_{{\textrm ve}}, \bar{t}_0)$, and data as random variables, an optimization problem of maximizing the mutual information (MI) between the data and model parameters is proposed. It is shown that uncertainty in recovered $k_{{\textrm PL}}$ from synthetic noisy data is reduced when optimal design parameters are considered; see section "Results".

In what follows, after defining some preliminary quantities, the mutual information between data and the model parameters is defined. Let ${\textrm z}\in {\textrm Z}= \mathbb {R}$, $\mathcal {P}\in \Theta \subset \mathbb {R}^3$, and $\mathcal {K}\in {\textrm D}\subset \mathbb {R}^{3{\textrm N}-1}$, where ${\textrm Z}, \Theta , {\textrm D}$ are data, model parameter, and design parameter spaces, respectively. It is remarked that mutual information are intractable and suffer from the curse of dimensionality as the model complexity is increased to consider more variables. The curse of dimensionality appears from the nested integrals inherent to the mutual information calculation. Further, mutual information calculations in this work utilizes the spatially invariant model while incorporating spatial variations in the mutual information through the parameter variance. For example, spatial variations in the T1 relaxation times among biological compartments are expected and are represented by the variance in the T1 parameters of our model. Our Bayesian approach explicitly accounts for modeling uncertainty including differences among biological compartments through the parameter variance.

#### Prior, likelihood, and evidence

Suppose $p_0(\mathcal {P})$ is the prior probability distribution function (PDF) of model parameters $\mathcal {P}$ and $p({\textrm z})$ is the PDF of the data ${\textrm z}$. Then the joint PDF $p({\textrm z}, \mathcal {P})$ must satisfy

9 $$\begin{aligned} p({\textrm z}, \mathcal {P}) = p({\textrm z}| \mathcal {P}) p_0(\mathcal {P}) = p(\mathcal {P}| {\textrm z}) p({\textrm z}), \end{aligned}$$

where $p({\textrm z}|\mathcal {P})$ is the conditional PDF of data conditioned on model parameters $\mathcal {P}$ (also referred to as the *likelihood* function) and $p(\mathcal {P}| {\textrm z})$ the conditional PDF of model parameters $\mathcal {P}$ conditioned on data ${\textrm z}$ (*posterior* of $\mathcal {P}$). The prior is taken as multi-variate Gaussian with mean $\mu {\mathcal {P}}$ and covariance matrix $\Sigma _\mathcal {P}$:

10 $$\begin{aligned} \mathcal {P}\sim p_0(\mathcal {P}) = \mathcal {N}(\mu {\mathcal {P}}, \Sigma _\mathcal {P}) = \frac{1}{2 \pi \det {\Sigma _\mathcal {P}}} \exp \left( - \frac{1}{2} \Vert \mu {\mathcal {P}} - \mathcal {P}\Vert ^2_{\Sigma ^{-1}_\mathcal {P}} \right) . \end{aligned}$$

Here $\Vert \mu {\mathcal {P}} - \mathcal {P}\Vert ^2_{\Sigma ^{-1}_\mathcal {P}} = (\mu {\mathcal {P}} - \mathcal {P})\cdot \Sigma ^{-1}_\mathcal {P}(\mu {\mathcal {P}} - \mathcal {P})$. Within this Bayesian setting, $\Sigma _\mathcal {P}$, is representative of biological variability. Within the scope of this manuscript, biological variability refers to potential patient specific differences in the pyruvate to lactate exchange rates, vascular tissue exchange rates, and bolus timing of the injected pyruvate.

To derive the expression for the likelihood function, first suppose that $\mathcal {G}= \mathcal {G}(\mathcal {K}, \mathcal {P})$ is the model prediction of data. Data and the model prediction are assumed to be related as follows:

11 $$\begin{aligned} {\textrm z}= \mathcal {G}(\mathcal {K}, \mathcal {P}) + \varepsilon \quad \Rightarrow \quad {\textrm z}- \mathcal {G}(\mathcal {K}, \mathcal {P}) \sim \mathcal {N}(0, \sigma _{\textrm z}) , \end{aligned}$$

where an additive model of noise is assumed and the noise, $\varepsilon$, is taken as Gaussian with a zero mean and standard deviation $\sigma _{\textrm z}$, i.e., $\varepsilon \sim \mathcal {N}(0, \sigma _{\textrm z}^2)$. Here, we assume that the the phase of the real and imaginary MR data acquisition is constant over time such that the signal may be phase corrected. The MR data acquisition is, in general, complex valued with real and imaginary components that are independent and Gaussian. However, within our phase corrected approach, only the real component is non-zero and Gaussian noise for the real channel is appropriate.

Therefore, the likelihood function $p({\textrm z}|\mathcal {P})$ takes the Gaussian form:

12 $$\begin{aligned} p({\textrm z}|\mathcal {P}) = \mathcal {N}(\mathcal {G}(\mathcal {K},\mathcal {P}),\sigma _{\textrm z}) = \frac{1}{2 \pi \sigma _{\textrm z}} \exp \left( - \frac{1}{2\sigma _{\textrm z}^2} \Vert \mathcal {G}(\mathcal {K},\mathcal {P}) - {\textrm z}\Vert ^2\right) . \end{aligned}$$

Here $|| \cdot ||$ denotes the Euclidean norm. Technically, $p({\textrm z}| \mathcal {P})$ is also conditioned on $\mathcal {K}$, but, for simplicity in notation, the dependence on $\mathcal {K}$ is suppressed.

With $p_0(\mathcal {P})$ and $p({\textrm z}|\mathcal {P})$ defined as above, the joint PDF $p({\textrm z}, \mathcal {P})$ can be computed using (9). Further, using (9), the PDF of data ${\textrm z}\in \mathbb {R}$ (evidence), $p({\textrm z})$, can be computed by marginalizing $p({\textrm z}, \mathcal {P})$ with respect to $\mathcal {P}$:

13 $$\begin{aligned} p({\textrm z}) = \int _{\Theta } p({\textrm z}, \mathcal {P}) d\mathcal {P}= \int _{\Theta } p({\textrm z}| \mathcal {P}) p_0(\mathcal {P}) d \mathcal {P}, \end{aligned}$$

where $\Theta$ is the parameter space.

#### Mutual information

Next, the statistical mutual information is defined and the optimization problem for design parameters $\mathcal {K}$ is posed. Given HP-MRI data, the accurate inference of pyruvate-to-lactate exchange rate parameters (and other parameters in $\mathcal {P}$) depends on the specific choice of control (design) parameters $\mathcal {K}$ as selection of $\mathcal {K}$ affects the information content in the measured data. The notion of mutual information^29^ provides a way to quantify the information content about the model parameters $\mathcal {P}$ in the data ${\textrm z}$. The mutual information between the two random variables *z* and $\mathcal {P}$ with PDFs $p({\textrm z})$ and $p_0(\mathcal {P})$ and the joint PDF $p({\textrm z},\mathcal {P})$ is defined as

14 $$\begin{aligned} I = I(\mathcal {K}) = \int _{\textrm Z}\int _\Theta p({\textrm z}, \mathcal {P})\ln \left( \frac{p({\textrm z}, \mathcal {P})}{p_0(\mathcal {P})p({\textrm z})}\right) d\mathcal {P}d{\textrm z}. \end{aligned}$$

Here, the mutual information *I* depends only on design parameters $\mathcal {K}$ and the forward model (1).

Using Bayes theorem, $p({\textrm z}, \mathcal {P}) = p({\textrm z}|\mathcal {P})p_0(\mathcal {P})$, it can easily be shown that

15 $$\begin{aligned} I(\mathcal {K})=\int _{\textrm Z}\int _\Theta p({\textrm z}|\mathcal {P})p_0(\mathcal {P})\ln \left( \frac{p({\textrm z}|\mathcal {P})p_0(\mathcal {P})}{p_0(\mathcal {P})p({\textrm z})}\right) d\mathcal {P}d{\textrm z}, \end{aligned}$$

or,

16 $$\begin{aligned} I(\mathcal {K}) =\int _{\textrm Z}\int _\Theta p({\textrm z}|\mathcal {P})p_0(\mathcal {P})\ln \left[ p({\textrm z}|\mathcal {P})\right] d\mathcal {P}d{\textrm z}- \int _{\textrm Z}p({\textrm z}) \ln p({\textrm z})d{\textrm z}H({\textrm z}; \mathcal {K}) - H({\textrm z}|\mathcal {P}; \mathcal {K}) , \end{aligned}$$

where the second term in the above equation is identified as information entropy $H({\textrm z}; \mathcal {K})$ and the first term as negative of the cross-information entropy, $H({\textrm z}|\mathcal {P}; \mathcal {K})$. That is

17 $$\begin{aligned} \begin{aligned} H({\textrm z}; \mathcal {K})&= - \int _{\textrm Z}p({\textrm z}) \ln p({\textrm z})d{\textrm z}, \\ H({\textrm z}| \mathcal {P}; \mathcal {K})&= -\int _{\textrm Z}\int _\Theta p({\textrm z}|\mathcal {P})p_0(\mathcal {P})\ln \left[ p({\textrm z}|\mathcal {P})\right] d\mathcal {P}d{\textrm z}. \end{aligned} \end{aligned}$$

**Optimization problem** In order to maximize the reduction in the uncertainty in the model parameters (i.e. to have the most confident estimates of the parameters $\mathcal {P}$), we propose to maximize the mutual information between the observation data and parameters of interest:

18 $$\begin{aligned} \max _{\mathcal {K}\in {\textrm D}} I(\mathcal {K}) = I(\mathcal {K}^*), \end{aligned}$$

where $\mathcal {K}^*$ is the design parameter for which *I* is maximum (assuming $\mathcal {K}^*$ exist).

Given a Gaussian prior and conditional probability of the data with respect to the parameters, the entropy of the data conditioned on the model parameter, i.e., $H({\textrm z}| \mathcal {P})$, is constant. Therefore, the optimization problem simplifies to

19 $$\begin{aligned} \mathcal {K}^*= \mathop {\mathrm {{\textrm arg max}}}\limits _{\mathcal {K}\in {\textrm D}} I(\mathcal {K}) = \mathop {\mathrm {{\textrm arg max}}}\limits _{\mathcal {K}\in {\textrm D}} H({\textrm z}; \mathcal {K}). \end{aligned}$$

#### Approximation of mutual information and information entropy

Mutual information calculations are computationally demanding due to high-dimensional integration over the parameter and data spaces. Combinations of both quadrature and sampling methods have been employed for mutual information calculations, each of which is well-suited to certain function classes^32–44^. These methods include Monte Carlo and Quasi-Monte Carlo methods^33–35^, lattice rules^36^, adaptive subdivision^37, 38^, neural network approximations^39^ and numerical quadrature^40^. Here the problem structure is used to accelerate computations and facilitate tractable numerical integration. Following^45^, Gauss-Hermite quadrature is applied in each dimension of mutual information integrals defined in (16) to numerically integrate multi-variate Gaussian random variables. Quadrature order is increased until convergence is observed. Finite difference time stepping is used to solve the ODE-based model presented in Sect. “The total signal”.

##### Automatic differentiation accelerated optimization for OED calculations

 For constant TR optimization, the auto-differentiation functions of MATLAB were used to calculate gradients of (16). In particular, design parameters $\mathcal {K}$ and state variables $\bar{\varvec{{\textrm c}}}$ were considered as optimization variables to minimize the objective function (16) with respect to the model constraints (1). Auto-differentiation provides the derivatives of the objective function and constraints with respect to this full space formulation. Given the derivatives in the full space formulation, the reduced space gradient of the objective function (16) with respect to the design parameters $\mathcal {K}$ may be calculated using an adjoint solve. For varying TR optimization, adjoint gradients were calculated by hand.

##### Inverse problem to recover rate parameter

 A MATLAB routine fmincon is used to solve the inverse problem of recovering model parameters $\mathcal {P}$ in the model (1) from the data generated in Sect. “Synthetic data” As an objective function for the inverse problem, square of the difference between data and model prediction of signals is used. Similarly, derivatives from the automatic differentiation feature in the MATLAB are utilized for numerical optimization.

The Cramér-Rao bound is computed to provide a reference for the uncertainty observed in the recovered $k_{{\textrm PL}}$.

$$\begin{aligned} \text {var}\left\{ k_{{\textrm PL}}\right\} \ge J^{-1}(\mathcal {P}) \end{aligned}$$

Here, *J* is the Fisher information matrix. Each time point of the HP signal evolution, Eq. (1), may be considered as an independent random variable, thus following^46^, the Cramér-Rao bound may be computed analytically with the Fisher information matrix given as follows.

$$\begin{aligned} J = \frac{\partial \textbf{m}}{\partial \mathcal {P}}^\top \Sigma ^{-1}_\mathcal {P}\frac{\partial \textbf{m}}{\partial \mathcal {P}} \qquad \textbf{m} = \left[ \bar{\varvec{{\textrm c}}}(t_1) \dots \bar{\varvec{{\textrm c}}}(t_{\textrm N}) \right] \end{aligned}$$

## Results

In this section, the main results of our analysis are presented. First, the optimal design parameters for different signal-to-noise ratios (SNRs) are shown. Optimal design parameters for both temporally constant and varying flip angles at each data acquisition are considered. Next, the reduction in uncertainty of $k_{{\textrm PL}}$ when using optimal design parameters generated synthetic data is shown.

### Optimized design parameters

As mentioned in Sect. “Mutual information based optimization of MR scan parameters”, multi-variate Gaussian is taken as a prior for uncertain model parameters, $\mathcal {P}= (k_{{\textrm PL}}, k_{{\textrm ve}}, \bar{t}_0)$. The mean and diagonal covariance matrix are fixed to

20 $$\begin{aligned} \mu _\mathcal {P}= (0.15, 0.05, 4), \qquad \Sigma _\mathcal {P}= {\textrm diag}(0.03, 0.01, 1.3) . \end{aligned}$$

For this choice of mean and variance, all quadrature points for a fifth order Gauss-Hermite quadrature approximation of numerical integration were positive. The remaining model parameters are fixed according to Table 1. Next, to fix the likelihood function, the Gaussian noise distribution, i.e., $\varepsilon \sim \mathcal {N}(0, \sigma _{\textrm z})$, is needed to be fixed. To consider reasonable values of $\sigma _{\textrm z}$, first the reference peak pyruvate signal ${{\textrm s}_{{\textrm P}}}_{{\textrm ref}}$ is calculated using the solution of the model with the default model and design parameters in Tables 1 and 2; it is found to be ${{\textrm s}_{{\textrm P}}}_{{\textrm ref}} = 0.6173$. Then for different signal-to-noise ratios (${\textrm SNR}$), the noise (standard deviation) in the individual signals, $\sigma _{\textrm s}$, and the standard deviation of the total signal, $\sigma _{\textrm z}$, are computed as follows, for ${\textrm SNR}\in \{2, 5, 10, 15, 20\}$,

21 $$\begin{aligned} \sigma _{\textrm s}({\textrm SNR}) = {{\textrm s}_{{\textrm P}}}_{{\textrm ref}} / {\textrm SNR}, \qquad \sigma _{\textrm z}({\textrm SNR}) = \sigma _{\textrm s}\sqrt{2{\textrm N}}, \end{aligned}$$

where ${\textrm N}$ is the total number of scans. For each ${\textrm SNR}$ and corresponding $\sigma _{\textrm z}({\textrm SNR})$ in the above list, optimal design parameters are obtained by solving the optimization problem (19). For simplicity, let $\mathcal {K}_{{\textrm OED}_{{\textrm SNR}}}$ denote the optimized design parameter corresponding to ${\textrm SNR}$ and $\sigma _{\textrm z}({\textrm SNR})$. The rationale behind considering different ${\textrm SNR}$ is that, in reality, data is expected to have varied signal-to-noise ratios and this is shown to impact the choice of optimal design parameters. The initial values of the design parameter $\mathcal {K}$ are listed in Table 2.

Two cases of optimization as described below are considered:

- *Constant flip angle optimization*. In this case, ${\textrm TR}_{k}$ is fixed to 3 seconds for all *k*, and the flip angles are assumed to be same for all scans. As a result, the optimization problem involved only two variables, $\theta _{{\textrm P}}$ and $\theta _{{\textrm L}}$.
- *Variable flip angle and TR optimization*. In this case, flip angles and TR values at all scans are optimized.

**Figure 1:**
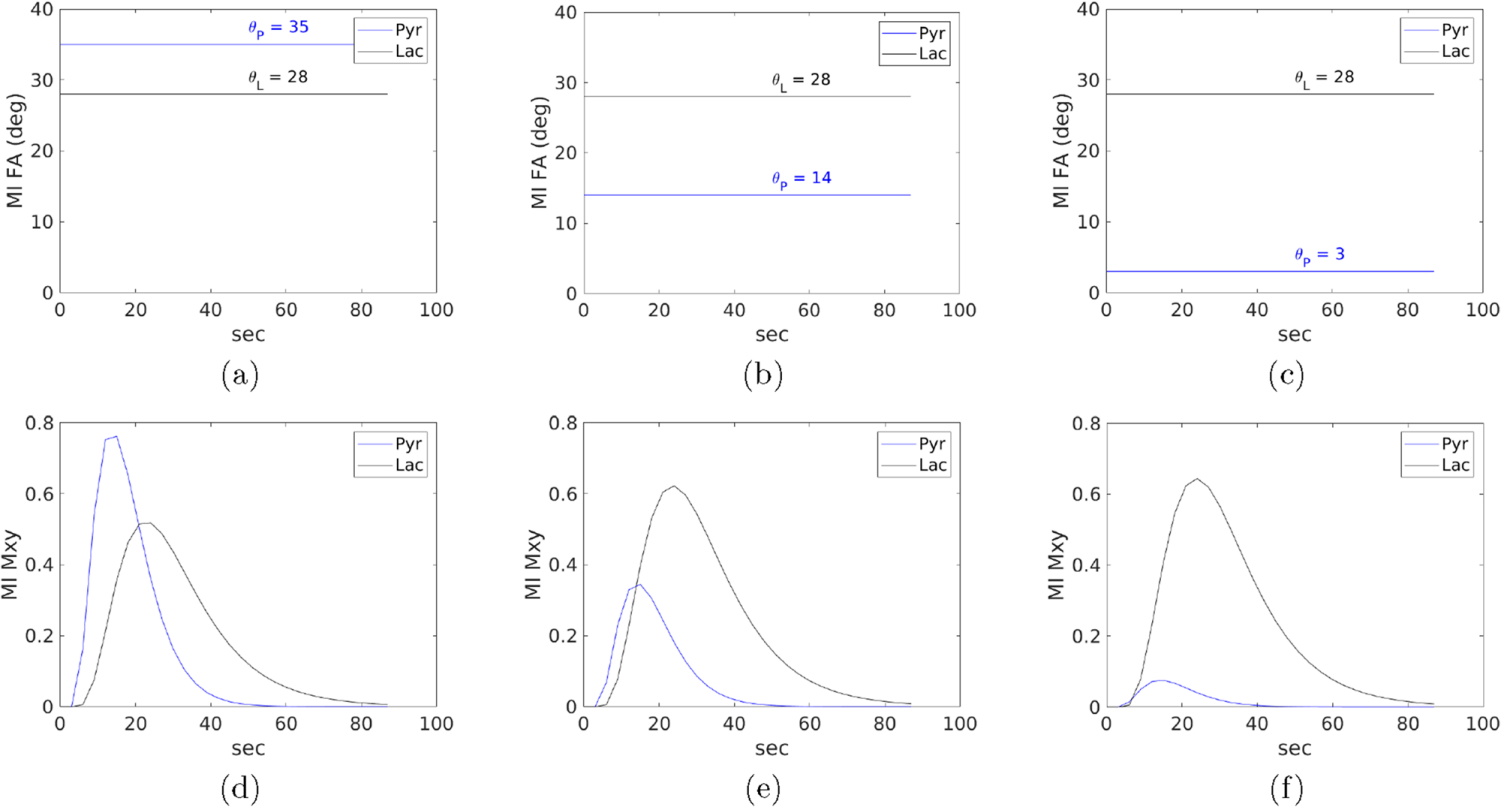
Optimized design parameters along with the signals of pyruvate and lactate obtained from the solution of the forward model using optimal design parameters. In (**a**), for noise $\sigma _{\textrm z}(2)$, i.e., $\sigma _{\textrm z}({\textrm SNR})$ for ${\textrm SNR}= 2$, the optimized flip angle scheme is shown for constant flip angles throughout the acquisition (optimal angles are $\theta _{{\textrm P}}^k = 35$ degrees and $\theta _{{\textrm L}}^k = 28$ degrees, for all $1\le k\le {\textrm N}$). The corresponding signal evolution of the transverse magnetizations () are shown in (**d**) for the constant flip angle case. Similarly, (**b**) and (**c**) present the optimized flip angles for ${\textrm SNR}= 10$ and ${\textrm SNR}= 20$; respectively. The corresponding signal evolution of the transverse magnetizations are shown in (**e**) and (**f**).

**Figure 2:**
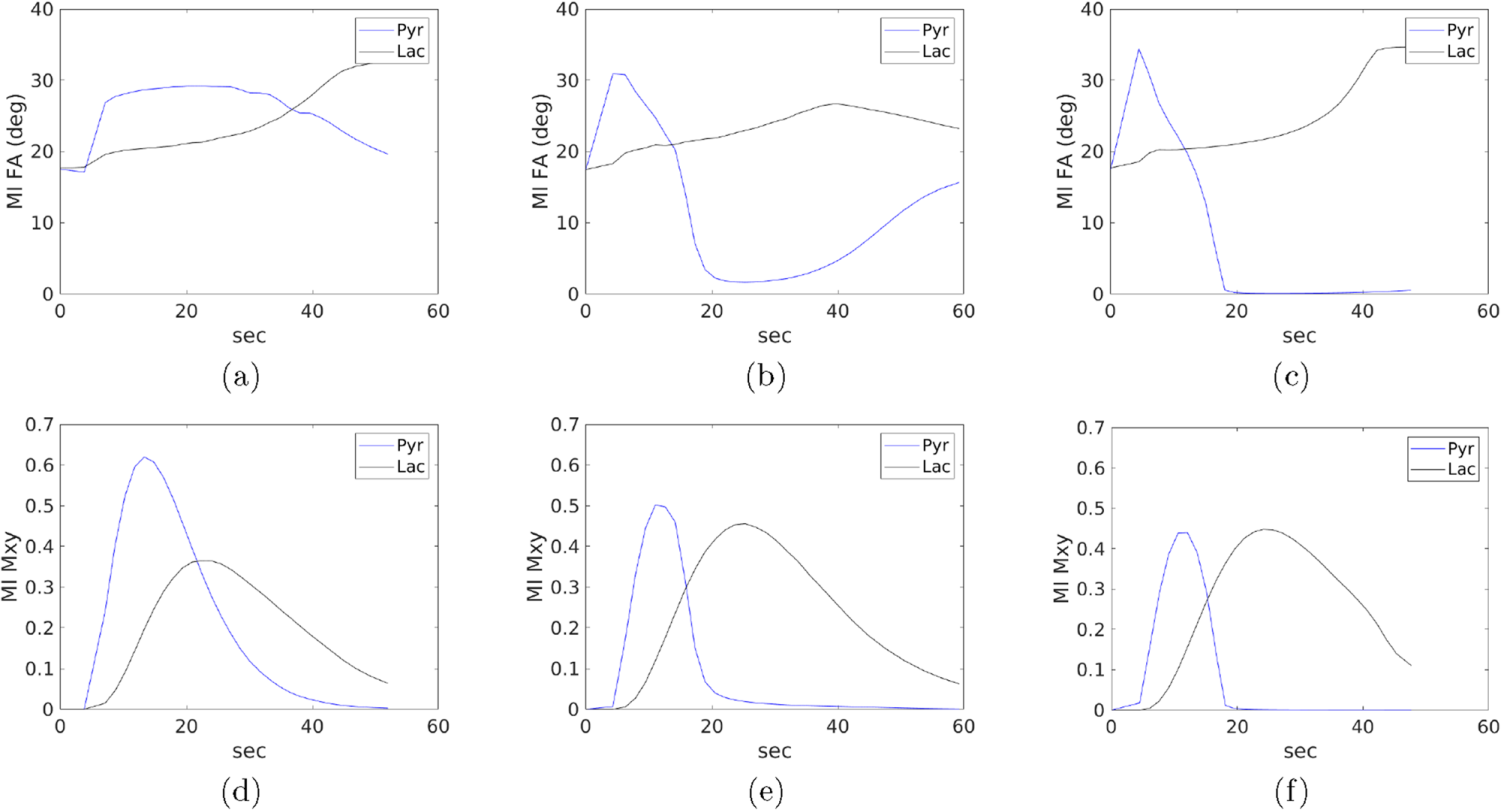
Optimized design parameters along with the solution of the forward model. In (**a**), for noise $\sigma _{\textrm z}(2)$, i.e., $\sigma _{\textrm z}({\textrm SNR})$ for ${\textrm SNR}= 2$, the optimizer considers jointly varying the flip angle and repetition time at each acquisition. The corresponding signal evolution of the transverse magnetizations () are shown in (**d**). Similarly, (**b**) and (**c**) present the optimized flip angles and repetition time for ${\textrm SNR}= 10$ and ${\textrm SNR}= 20$, respectively. The corresponding signal evolution of the transverse magnetizations are shown in (e) and (f). Note here that x-axis in all plots is for time and therefore the plots implicitly include the values of optimized ${\textrm TR}_k$, $1\le k \le {\textrm N}$.

**Figure 3:**
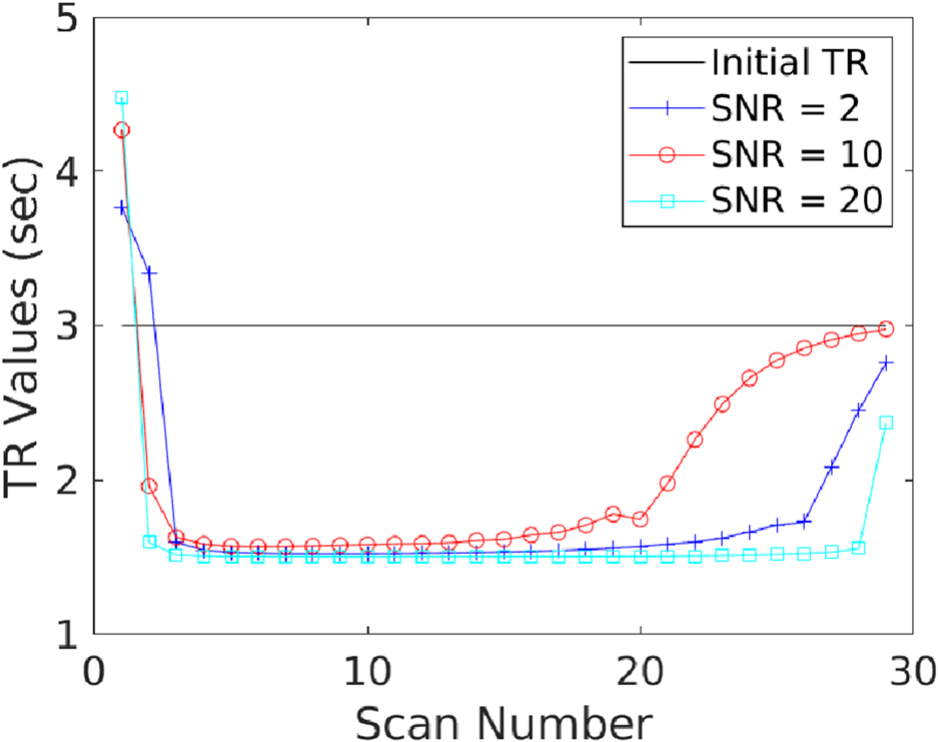
Plot of optimal repetition times for three cases of ${\textrm SNR}\in \{2, 10, 20\}$.

For the case of constant flip angle optimization, the optimized design parameters, $\mathcal {K}_{{\textrm OED}_{{\textrm SNR}}}$, corresponding to the five SNRs are tabulated in Table 3. Figs. 1 and 2 represent the optimal design parameters, $\mathcal {K}_{{\textrm OED}_{{\textrm SNR}}}$, for ${\textrm SNR}= 2$, ${\textrm SNR}= 10$, and ${\textrm SNR}=20$ for the two cases of optimization problems, respectively. Fig. 1 presents the optimal solution when considering a fixed repetition time of 3s and optimizing for pyruvate and lactate flip angles that are constant in time. Fig. 2 presents the optimal solution when allowing the repetition time and flip angles to vary at each acquisition for a fixed number of data acquisitions, ${\textrm N}$. The optimal values of design parameters are shown in Fig. 1(a-c) and Fig. 2(a-c). The time varying optimized design parameters are significantly different from the constant value flip angle scheme. Fig. 1(d-f) and Fig. 2(d-f) show the transverse magnetizations for ${\textrm s}^k_{\textrm P}, {\textrm s}^k_{\textrm L}$. For the case of variable design parameters, optimized ${\textrm TR}$ values are shown in Fig. 3.

**Table 3:** Optimized design parameters considering constant flip angles throughout the scan. Repetition time is fixed to ${\textrm TR}_k = 3$ s, for all *k*.

${\textrm SNR}$	$\theta _{{\textrm P}}$ (degrees)	$\theta _{{\textrm L}}$ (degrees)	${\textrm TR}$ (s)	${\textrm SNR}$	$\theta _{{\textrm P}}$ (degrees)	$\theta _{{\textrm L}}$ (degrees)	${\textrm TR}$ (s)
2	35	28	3	5	35	28	3
10	14	28	3	15	4	28	3
20	3	28	3				

### Validation of the uncertainty reduction using optimal design parameters

The basic workflow in verification includes generating the samples of noisy data associated with different design parameters following Sect. “Synthetic data” and then solving the inverse problem to recover uncertain model parameters for each sample of noisy data. From the recovered $k_{{\textrm PL}}$ for different samples of data, the mean and the standard deviation is computed. Specifically, the standard deviation is used as a measure of the uncertainty in the recovered $k_{{\textrm PL}}$.

**Figure 4:**
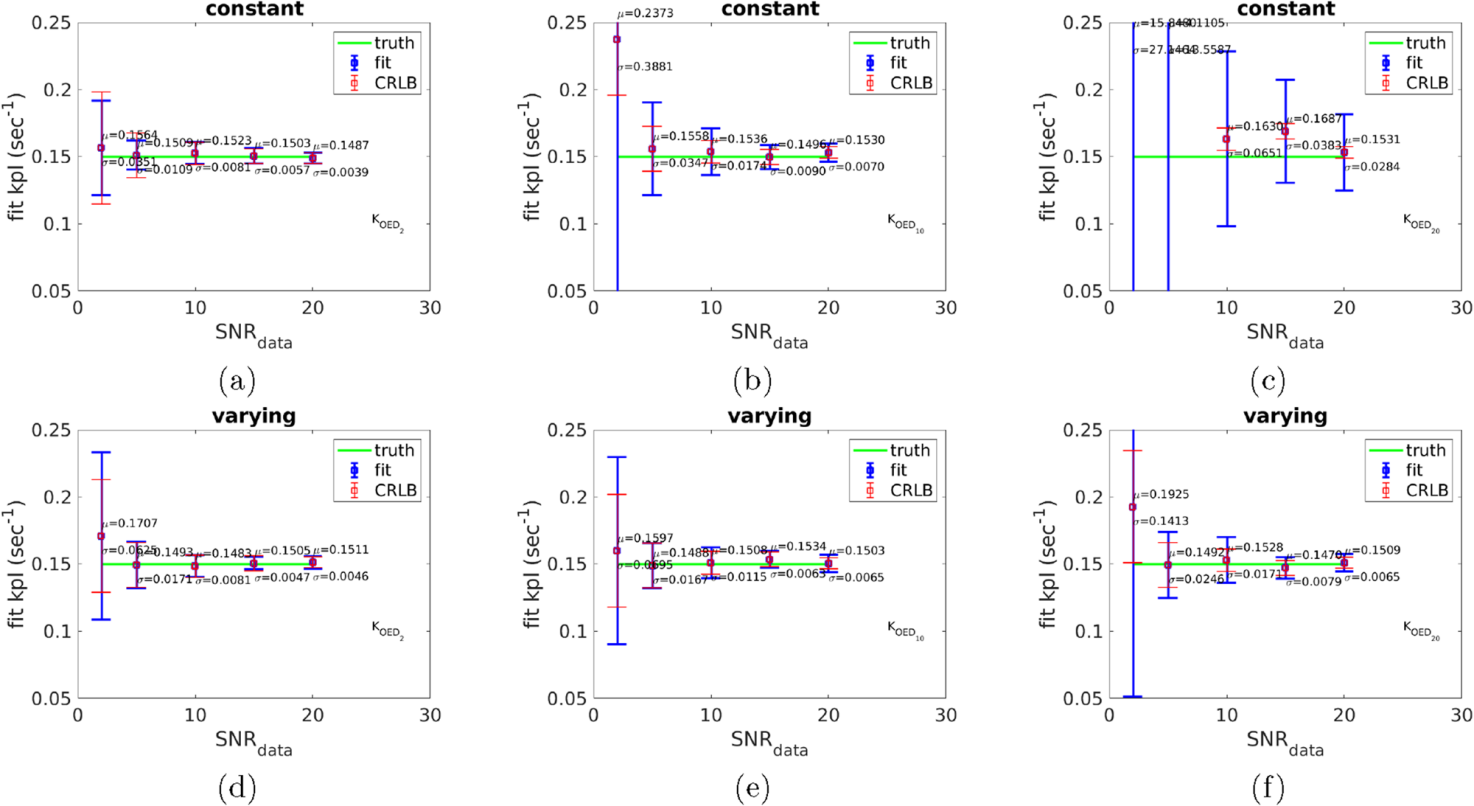
Plot of inferred $k_{{\textrm PL}}$ from 25 samples of noisy data based on the synthetic data from the solutions of the model in (1). The x-axis is the value of ${\textrm SNR}_{{\textrm data}}$ employed in computing noisy data. (**a**) and (**d**) correspond to $\mathcal {K}_{{\textrm OED}_{2}}$ for a constant and varying flip angle and TR scheme, respectively. (**b**) and (**e**) correspond to $\mathcal {K}_{{\textrm OED}_{10}}$ for a constant and varying flip angle and TR scheme, respectively. (**c**) and (**f**) correspond to $\mathcal {K}_{{\textrm OED}_{20}}$ for a constant and varying flip angle and TR scheme, respectively.

**Figure 5:**
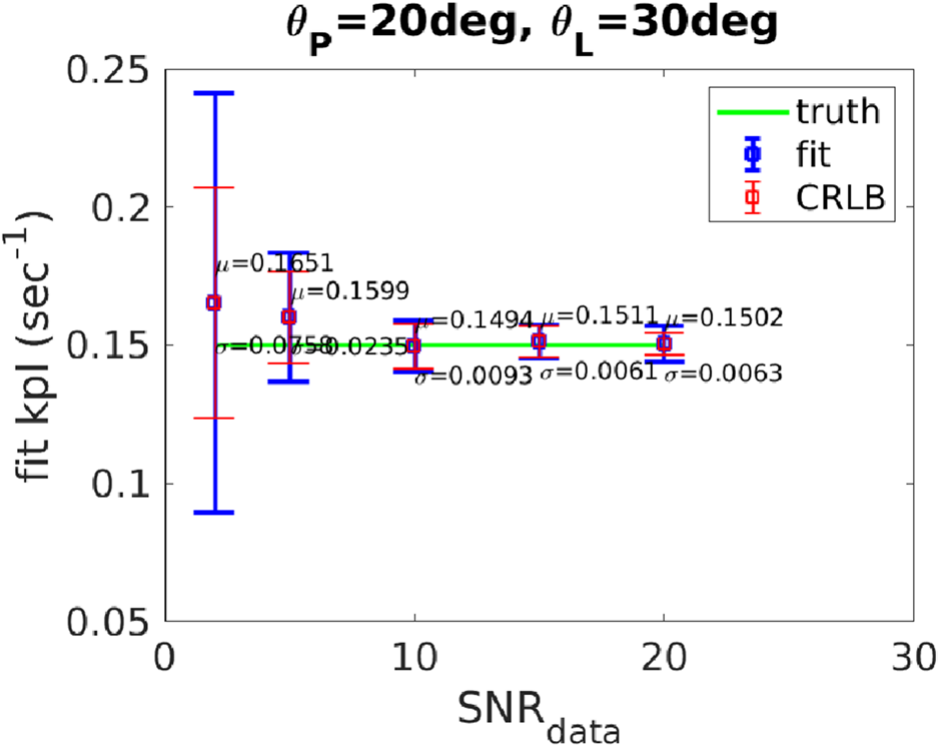
Plot of inferred $k_{{\textrm PL}}$ from 25 samples of noisy data based on the synthetic data from the solutions of the model in (1). The x-axis is the value of ${\textrm SNR}_{{\textrm data}}$ employed in computing noisy data. Here, the accuracy and precision obtained for flip angles are similar to values currently used in our human studies, $\theta _{{\textrm P}}^k=20$ and $\theta _{{\textrm L}}^k=30$ is shown as a control for Fig. 4.

First using the model in (1), data ${\textrm Y}_{{\textrm OED}_{{\textrm SNR}}}$ (for ${\textrm SNR}= 2, 5, 10, 15, 20$), corresponding to $\mathcal {K}_{{\textrm OED}_{{\textrm SNR}}}$, is obtained. Then the uncertain parameters $\mathcal {P}= (k_{{\textrm PL}}, k_{{\textrm ve}}, \bar{t}_0)$ in the model are recovered from the 25 samples of noisy data. The remaining model parameters are drawn from the Table 1. In Fig. 4, the statistics (mean and standard deviation) of recovered $k_{{\textrm PL}}$ corresponding to optimal design parameters shown in Figs. 1and 2 is presented. The SNR (SNR to generate noisy data) along the x-axis corresponds to the value of $\sigma _{\textrm s}({\textrm SNR}_{{\textrm data}})$ added to the data ${\textrm Y}_{{\textrm OED}_{{\textrm SNR}}}$ to generate noisy data; see (8) for the definition of noisy data. The y-axis represents the mean and standard deviation of the $k_{{\textrm PL}}$ recovered from the inference. The known value of $k_{{\textrm PL}}$ used to generate the noise corrupted data is shown as a horizontal line at $y = 0.15$ for a reference. Fig. 4(a) and Fig. 4(d) correspond to $\mathcal {K}_{{\textrm OED}_{2}}$ for a constant and varying flip angle scheme; respectively. Fig. 4(b) and Fig. 4(e) correspond to $\mathcal {K}_{{\textrm OED}_{10}}$ for a constant and varying flip angle scheme; respectively. And finally Fig. 4(c) and Fig. 4(f) correspond to $\mathcal {K}_{{\textrm OED}_{20}}$ for a constant and varying flip angle scheme; respectively. The Cramér-Rao bound is provided as a reference for the lower bound of the variance in each. Fig. 5 provides a control for the accuracy and precision obtained for flip angles similar to those currently in use in our clinic^47, 48^, $\theta _{{\textrm P}}^k=20$ and $\theta _{{\textrm L}}^k=30$. Similarly, the Cramér-Rao bound is provided as a reference for the lower bound of the variance. The results show that the uncertainties in recovered parameter using $\mathcal {K}_{{\textrm OED}_{2}}$ are comparable to the current clinical pulse sequence implementation. In fact, $\mathcal {K}_{{\textrm OED}_{2}}$ demonstrates improved performance for all ${\textrm SNR}_{{\textrm data}}$ except ${\textrm SNR}_{{\textrm data}} = 20$.

Generally, an improvement in the accuracy and precision of the recovered parameter is seen with increasing ${\textrm SNR}_{{\textrm data}}$. A time-varying flip angle scheme leads to a higher parameter optimization that can further improve the quantitative value of mutual information over a constant flip angle scheme. However, the constant flip angle scheme, 35 and 28 degrees for pyruvate and lactate measurements, leads to the accuracy and precision comparable to the variable flip angle schemes obtained from our method.

## Discussion

The evaluation of accuracy and precision as a function of pulse sequence design is effectively a bi-level optimization problem where the goal is to solve two nested optimization problems: (1) find the pulse sequence that produces the best accuracy and precision for the (2) best curve fit to the data. Direct numerical optimization of the bi-level cost functions(s) is challenging^49^. The mutual information objective function of this study as well as signal maximization and Fisher information in the literature^15, 18, 19^ are effectively proposing a surrogate objective function as a numerically tractable approximation to the bi-level optimization problem of interest. Within the mutual information based optimal experimental design formulation, a time varying flip angle and repetition time scheme is seen to provide significant differences in the pulse sequence as compared to the case when excitation angles are fixed to a constant value over time with a fixed repetition time of 3 s. Indeed, the varying flip angle and repetition time scheme leads to a higher dimensional parameter optimization that provides more degrees of freedom to further improve the quantitative value of mutual information over the constant flip angle scheme. However, as seen in Fig. 4, the constant flip angle scheme leads to comparable accuracy and precision when considering the inference from noise-corrupted data. The time varying scheme is seen to be more sensitive to noise corruption of the expected signal and is generally seen to have the higher variance in the parameter recovery at lower ${\textrm SNR}_{{\textrm data}}$. The mutual information calculations are generally seen to achieve the Cramér-Rao bound with higher SNR.

The purely theoretical nature of these results is a limitation of the study. However, the two-compartment model analyzed in this work is utilized in numerous studies^50–56^ and, when parameterized with physically meaningful values of the parameters, good data agreement is shown within these studies. Results of this manuscript are thus relevant towards guiding future data collection.

The reduction in the recovered variance is seen to be correlated with the assumed noise value added to the data. Intuitively, less noise resulted in less variance in the parameter recovery. Less intuitively, the optimal MI solutions for flip angles are seen to vary with the noise value of the signal conditional probability model $p({\textrm z}| \mathcal {P})$. The greatest reduction in measurement uncertainty is seen for the MI optimal solution corresponding to low SNR of the signal conditional probability model. Here, the lower flip angle is applied to the non-injected secondary substrate and higher pyruvate signal is maintained throughout the acquisition. This could be due to the system being so signal limited for the low SNR case that it is forced to leverage the pyruvate signal to extract any additional information about the metabolic exchange rate. The excitation angles optimized for the higher SNR condition reduce the pyruvate excitation angle to save magnetization for subsequent conversion while simultaneously increasing the lactate flip angle. Within the time varying optimization, the pyruvate excitation angle is reduced to zero after 20 s. For high SNR this suggests that the lactate signal is sufficient to accurately determine the metabolic exchange rate and measuring a large pyruvate signal after the initial bolus is less important.

This work considers uncertainty in the vascular-tissue exchange parameter, bolus arrival time, and rate constants modeled through a Gaussian prior. However, a more comprehensive evaluation of additional uncertain parameters would further evaluate the stability of our results. Additionally, the effect of alternative prior formulations such as uniform distributions for the prior parameters may also be investigated. The numerical computation in this work is also limited by the quadrature scheme for numerical integration of the mutual information integrals. Adding additional sources of uncertainty suffers from the well-known curse of dimensionality^57^ and alternative integration schemes such as Markov chain Monte Carlo may be more effective.

Further, the current approach considers the real component of the readout and assumes SNR such that Gaussian statistics is an appropriate noise model for the signal acquisition. Rician statistics^58^ is known to be more appropriate as the noise model for low SNR and the low SNR range is expected to be more important toward the end of the HP data acquisition as the signal decays. Rician statistics will be considered in future efforts to optimize acquisition parameters at low SNR or when considering both the real and imaginary components of the signal magnitude.

Alternative to the spatial-invariant model, high-fidelity models may also be considered to determine optimal design parameters and to recover model parameters from the data. However, for such an approach to work, a realistic high-fidelity model is needed keeping in mind the major factors in HP-MRI physics. Additional model fidelity may include permutations of lactate and pyruvate that are endogenous as well as hyperpolarized. Intravascular, extracellular, and intracellular species may also be considered. Nonlinear pyruvate-to-lactate conversion parameters such as from a Michaelis-Menten relationship may also be considered. Additional formulations may also consider the impact of blood flow in the simulations directly though Dirichlet boundary conditions, as convective transport through porous media^59^, or as a sophisticated 3D-1D coupling with vasculature treated as 1D curvilinear segments^60, 61^.

In summary, our results suggest that the constant flip angle scheme corresponding to $\mathcal {K}_{{\textrm OED}_{2}}$ is the best choice in terms of accuracy and precision of the parameter recovery. Results at $\mathcal {K}_{{\textrm OED}_{2}}$, $\theta _{{\textrm P}}^k=35$ and $\theta _{{\textrm L}}^k=28$, are comparable to the current clinical pulse sequence implementations, $\theta _{{\textrm P}}^k=20$ and $\theta _{{\textrm L}}^k=30$, and demonstrate an improved performance at low ${\textrm SNR}_{{\textrm data}}$. Further, the constant flip angle scheme may represent a practical choice for implementation on the pulse sequence hardware.

## Data Availability

The codes and relevant data files to reproduce the results will be publicly available in the following GitHub repository: https://github.com/prashjha/HyperpolarizedMRI.
